# Comparison of Visual Skills between Federated and Non-Federated Athletes

**DOI:** 10.3390/ijerph20021047

**Published:** 2023-01-06

**Authors:** Miguel Ángel Sánchez-Tena, Xabier Rodríguez-Alonso, Clara Martinez-Perez, José Francisco Tornero-Aguilera, Vicente J. Clemente-Suárez, Celia Sanchez-Ramos, Cristina Alvarez-Peregrina

**Affiliations:** 1 Department of Optometry and Vision, Faculty of Optics and Optometry, Universidad Complutense de Madrid, 28037 Madrid, Spain; 2 ISEC LISBOA—Instituto Superior de Educação e Ciências, 1750-179 Lisbon, Portugal; 3 Faculty of Sports Sciences, Universidad Europea de Madrid, 28670 Madrid, Spain; 4 Studies Centre in Applied Combat (CESCA), 45007 Toledo, Spain; 5 Grupo de Investigación en Cultura, Educación y Sociedad, Universidad de la Costa, Barranquilla 00928-1345, Colombia; 6 Grupo de Investigación en Visión y Oftalmología, Universidad Complutense de Madrid, Avda, Arcos de Jalón 118, 28037 Madrid, Spain

**Keywords:** sports vision, motor performance, oculomotor behavior

## Abstract

Background: To perform motor tasks, athletes must gather a considerable amount of visual information quickly. Evidence shows that visual skills vary between athletes and non-athletes, and impact athletic performance. However, there is no scientific evidence suggesting that there are any differences between the visual skills of federated and non-federated athletes. As such, the objective of this paper was to compare how visual skills influence the sports performance of federated and non-federated athletes, respectively. Methods: A visual examination has been conducted on a total of 52 athletes between 18 and 37 years of age. The COI-Sport Vision system screen (International Optometry Center, Madrid, Spain) was used to examine static visual acuity, dynamic visual acuity, contrast sensitivity, stereopsis, fixation disparity, visual memory, identification, anticipation time, peripheral awareness, and hand-eye coordination. Results: On average, federated athletes train more hours per day than non-federated athletes (1.4 ± 0.8) (*p* = 0.046). A significant correlation was observed between the average time of visual memory (β = −0.0683, *p* < 0.001), the average time of anticipation (β = 0.006, *p* = 0.009), the average time of peripheral awareness (β = 0.026, *p* = 0.002), hand-eye coordination (β = 0.028, *p* = 0.004), dynamic visual acuity (β = 0.055, *p* < 0.001), and the number of training hours. Conclusion: Results suggest that federated athletes are more concerned about their ocular health. Nonetheless, no differences were found in the oculomotor skills of both groups. Further investigation is required to consider each sport discipline individually.

## 1. Introduction

Sport in Europe is organized in a system of national federations in which only the major federations (usually one per country) are associated with the European and international federations. The European federations form the top of the pyramid, and one national federation from each country is permitted to be a member. The European federations are responsible for imposing sanctions on athletes who take part in any championships that are not recognized or authorized by the international federation. The national federations are on the second level of the pyramid, and there is only one national federation per discipline, therefore giving them a monopolistic position. These national federations are comprised of regional federations, and they are responsible for regulating all general matters within their discipline. The national federation for each sport is the only body authorized to organize recognized championships within the country. Regional federations are on the third level of the pyramid, comprised of local sports clubs. Regional federations are responsible for organizing regional championships and for coordinating sports on a regional level. Clubs form the foundation of the pyramid, and these offer the possibility for anyone to engage in sports, encouraging the development of new generations of athletes [1].

In recent years, the lack or loss of motivation, as well as the lack of time and the onset of sport-related injuries has resulted in a large number of younger athletes giving up participation in federated sports, choosing instead to engage in non-federated sports or even give up the sport altogether [2]. As a general rule, athletes involved in federated sports are required to demonstrate greater continuity throughout the season. Likewise, the amount of effort and commitment demanded of them, both in terms of training and participation in competitions, is much higher and more intense than that expected of athletes who participate in non-federated sports. Isorna Folgar et al. [3] observed that athletes who a sports organization federated had greater intrinsic and extrinsic motivation, favoring their continuity in sports. However, Prieto [4] found that federated runners presented lower scores regarding self-confidence, achievements of personal goals, self-esteem, and search for recognition. Nonetheless, they had a superior lifestyle, given that they considered a sport a part of their lives and not just a hobby.

To perform motor tasks appropriately, athletes must quickly gather considerable visual information [5]. There is evidence that visual skills vary between athletes and non-athletes; likewise, it has been suggested that visual skills can impact athletic performance. Boden et al. [6] compared the depth perception between baseball players and non-athletes, observing that baseball players boast better visual skills than their non-athlete counterparts. The results attained in a recent study by Vera et al. [7] were also in line with those observed in the study above, with basketball players demonstrating better visual skills than players without a sports background. However, it is unclear whether we should consider visual skills an innate talent, or whether these skills are enhanced through sports practice. It has been suggested that can improve certain visual skills through continuous practice and the introduction of sports vision training programs; however, innate contributions seemed insignificant [8].

Vision training has also been found to have an impact on injury prevention. For both athletes and coaches, preventing injuries that can threaten their long-term careers is paramount. Therefore, research has focused on the neurological sequelae of sports-related concussions, mainly, in the prevention of traumatic brain injuries due to sports [9].

Vision training is currently being studied to prevent injuries, especially head trauma. Brain training is thought to help prevent concussions. This is because the player is more aware of the pitch. In addition, it also helps in better recovery after a concussion [9]. Various studies have shown that sports performance can be improved through vision training programs and methods, and injuries can be prevented [8,10,11,12,13,14]. These studies were conducted in various sports (baseball, football, softball, soccer, basketball, and table tennis), as well as at various sports levels, from high school to elite professionals. Concluding that, by implementing visual training within sports training, an improvement in performance and prevention of injuries is obtained. This increases the value of the sports team, that is, athletes, coaches, and medical personnel.

To date, there is no scientific evidence to suggest any differences between the visual skills of federated and non-federated athletes. As such, the objective of this paper was to compare how visual skills influence the sports performance of federated and non-federated athletes, respectively.

## 2. Materials and Methods

This observational, descriptive, prospective study was conducted between October and December 2021. A visual assessment was performed on university athletes aged 18 to 37 years. The athletes were volunteers from the European University of Madrid federated university teams. The players in the sample of this study were men and women, all federated and belonging to university sports schools of basketball and volleyball.

The digital COISport Vision system (Centro de Optometría Internacional, Madrid, Spain) was used to assess the participants’ vision. This software has been developed to provide a testing battery to evaluate several visual, cognitive, and sensorimotor skills to generate a performance profile [15]. A 50-inch screen was used. The distance to carry out the test varies between 0 and 6 m.

The vision assessment was comprised of the following tests:

- Static visual acuity: The ability of the visual system to distinguish the details of a static object. It was performed at a distance of 6 m with the contrast at 100%. The test used to measure static visual acuity was Snellen’s E.
- Dynamic visual acuity: Minimum identifiable size of a moving object. Optotypes of letters with 100% contrast were presented. The movement was with an angular speed of 32 rpm. The computerized Wayne Rotator robot was used. The subject had to stand at a distance of 6 m. Letters and numbers are printed on the panel in front of the patient. The patient has to go to the line that begins with the letter “O” and then to the one that begins with the letter “F”.
- Contrast sensitivity: The ability of the visual system to distinguish an object from the background. The luminosity contrast sensitivity function test was used. The patient was located 3 m from the screen. On the screen are circles formed by bars of different and frequent contrasts (contrast bars from 4% to 0.10% and in frequencies of 1.5/3/6/12/18 cycles/degree). The patient should say whether the bars are oriented vertically, slightly to the right, or slightly to the left.
- Stereopsis: Ability to integrate the images received through each eye into a single three-dimensional image, in relief, and with depth. The purpose of the program is to determine the minimum stereoscopic disparity that the subject can detect in the second arc. The patient stands at a distance of 3 m from the screen.
- Fixation disparity: Slight difference between the two points of view provided by both eyes. To measure fixation disparity, a red cross and a green arrow were used. The vertical and horizontal disparity of all subjects is obtained by aligning the arrow with the cross, both vertically and horizontally. Subjects stand at a distance of 3 m from the screen and wear red/green filter glasses wore red/green filter glasses (the red filter was always worn in front of the directing eye). The deviations obtained were measured, in prismatic diopters.
- Visual memory: Ability to recall visually received information. The tic-tac-toe test was used. The subject is presented with a grid divided into nine spaces, for one-tenth of a second, with several repetitions. Five of these spaces are randomly occupied by symbols. Then the subject, at a distance of 0.5 m from the screen, must mark the location of the symbols as quickly as possible.
- Identification: Ability to identify one stimulus among several confusion-inducing stimuli. The subject located 0.5 m from the screen, has to touch the red ball that moves across the screen. Along with the red ball, several different colored balls appear and move around on the screen to distract the subject. The results of accuracy are given by sectors.
- Anticipation time: Ability to accurately predict the moment a moving object reaches a given position, speed, and direction. A red ball moves across the screen. The subject, located 0.5 m from the screen, has to touch the screen just as the ball passes through a rectangular area in any position on the screen. The goal is for the subject to be able to predict when the ball is going to pass through the rectangle, pressing with their hands or feet on the sensors to stop it. The test was performed with the dominant hand.
- Peripheral awareness: The ability to see objects in your field of vision, but are not in the direct line of vision. The character test was used. In this test, one of the characters presented differs from the rest. The results are recorded on the percentage of accuracy and the mean response time. The test is carried out at a distance of 0.5 m between the subject and the screen.
- Hand-eye coordination: Ability to perform activities in which the eyes and hands are used simultaneously. The directional arrows test was used. The arrows are presented sequentially so that they point in different directions. The subject, located at a distance of 0.5 m from the screen, has to touch the arrow directly on the screen before the arrow changes its direction. It is important to note execution speed since the number of correct answers and the execution time is recorded.

In addition, all participants completed a survey about their training and performance.

The research described here adhered to the Declaration of Helsinki and was approved by the ethics investigation committee of Universidad Europea de Madrid (CEI-UE) under the code CIPI/21/19-05.

### Statistical Analysis

Statistical analysis was conducted using the SPSS 25.0 software (SPSS Inc., Chicago, IL, USA). The normal distribution of the variables was conducted using the Kolmogorov-Smirnov test, with a significance level equal to 0.05. As a result of a non-parametric distribution, the non-parametric Kruskal Wallis or U-Mann-Whitney tests (quantitative variables), and the Chi-square test (qualitative variables) were used to determine whether or not there were any differences between the groups. Passing-Bablok linear regression analysis was used to analyze the association between continuous quantitative variables. In the regression analysis, the factors associated with the number of training hours were analyzed, visual memory, identification, visual acuity, fixation disparity, anticipation time, peripheral awareness, and hand-eye coordination as dependent variables.

## 3. Results

The total sample comprised 53 athletes in the visual screening. The participants were classified into two groups: federated and non-federated athletes. Thus, the sample was divided into 18 (34.6%) federated athletes and 34 (65.4%) non-federated athletes.

### 3.1. Demographic Analysis

The average age of participants was 22.7 ± 4.1 years old, and the median age [IQR] was 21 [3]. Regarding this, 65.4% of the participants (n = 34) were male and 34.6% (n = 18) were female. No significant differences were found in age (*p* = 0.116), gender (*p* = 0.761), or affiliation to a federation.

Table 1 shows the demographic data in detail.

### 3.2. Survey on Systemic Diseases and Accidents

Table 2 shows the data retrieved from a survey completed by the group of athletes who were required to give Yes or No answers when asked a series of questions related to systemic diseases and accidents/injuries.

### 3.3. Survey on Their Sports Performance

On average, federated athletes, that is to say, athletes who are affiliated with a sports federation (1.8 ± 0.8), train more hours per day than non-federated athletes (1.4 ± 0.8) (*p* = 0.046). However, when asked whether their performance was better during the day or at night, there were no significant differences between the answers given by federated and non-federated athletes (*p* = 0.064).

Table 3 shows the data retrieved from a survey completed by a group of athletes in which they were required to give Yes or No answers when asked a series of questions related to their sports performance.

### 3.4. Survey on Vision

Table 4 shows the data retrieved from a survey completed by a group of athletes in which they were required to give Yes or No answers when asked a series of questions about the use of glasses and the presence of ocular symptoms.

### 3.5. Visual Assessment

Table 5 shows the visual test values and the significant differences between federated and non-federated athletes. When applying Passing-Bablok regression, a significant correlation was observed between the average time of visual memory (β = −0.0683, *p* < 0.001), the average time of anticipation (β = 0.006, *p* = 0.009), the average time of peripheral awareness (β = 0.026, *p* = 0.002), hand-eye coordination (β = 0.028, *p* = 0.004), dynamic visual acuity (β = 0.055, *p* < 0.001) and the number of training hours.

## 4. Discussion

This is the first study to compare how visual skills affect the on-field performance of federated and non-federated athletes. Based on the understanding that federated athletes tend to perform at a higher level, this study demonstrated that they were more satisfied with their performance than non-federated athletes.

No significant differences were found between federated and non-federated athletes regarding whether or not they perform ocular warm-up exercises before competing. This may be because sports vision remains a rather new specialization, largely unknown by most athletes and coaches. It is worth mentioning that to reduce the risk of injury, just as athletes undertake muscular warm-ups, they should also gradually increase their eye efforts through a warm-up to ensure that their internal organs are functioning properly by matching the effort and movement made. As a result, and to decrease the risk of injuries, it is important to prepare the eyes for the anticipated physical effort [16].

Regarding visual symptoms, no significant differences were recorded between the two groups. However, more non-federated athletes mentioned problems related to blurred vision than their federated peers. The results attained in this study were in line with those obtained by Jorge J et al. [17] who found that elite athletes use means of vision correction if needed. Therefore, this suggests that elite and federated athletes are more concerned about their visual health and consider that their performance can be improved by using visual correction methods.

Most studies have found that professional athletes have better visual skills than non-professional athletes [8,18,19]. This paper demonstrated that players have better monocular static and dynamic visual acuity and better contrast sensitivity at higher frequencies. Our results matched those obtained in the studies by Vera et al. [7] and Paulus et al. [20] who confirmed that there were no differences between both groups in terms of the results attained in the stereopsis or accommodation tests. However, differences were recorded in the results attained for other skills, for example in the binocular tests. There are two possible reasons for this disparity. Firstly, as Paulus et al. [20] stated, stereopsis tests are not sensitive enough to reveal differences between groups, therefore suggesting the need for the methodology used, both in this test and in any others, to be further developed, and, likewise, a standardized protocol must be established to analyze visual skills in sports. Secondly, it is important to consider that some skills are developed more in specific sports. For example, stereopsis and ocular motions are hardly developed in basketball players [7,21].

The possible cause of the differences in static and dynamic visual acuity between federated and non-federated athletes may be that federated athletes are normally professional athletes. Thus, as demonstrated by Prieto et al. [4], professional players, given their extensive experience, integrate visual information with the sporting experience, so they encode the information through a more complex visuomotor integration system.

Therefore, as most of the athletes in our study play ball sports, differences were found in dynamic visual acuity. As observed in the study by Uchida et al. [22] these athletes are better able to detect moving objects and, likewise, have a more developed dynamic visual acuity given the increased number of training hours.

Concerning oculomotor skills, no differences were found between the two groups. The reason for not finding differences is that oculomotor skills vary depending on the sport. This contradicts the results of other studies [18,23,24,25] that found some differences. However, it is worth noting that in the studies conducted by Millard et al. [26] and Chase et al. [27], no differences were found in terms of visual memory. In addition, the studies conducted by Nascimento et al. [28,29] did not report any differences in anticipation time, hand-eye coordination, identification, peripheral awareness, and visual memory.

Although there are many reasons for the lack of similarity in the results obtained, one of the reasons may be the inclusion of athletes from a diverse range of sports disciplines taking into account the fact that some skills are more developed by athletes in certain disciplines than in others. In addition, even though federated athletes train for longer their training sessions are more intense. All participants play sports regularly, meaning that this difference would not affect the development of such skills.

Further research must be conducted into this topic to analyze the difference between the visual skills of federated and non-federated athletes, considering the sports discipline they are involved in. The studies have focused on examining the psychological factors between both groups, which might help develop visual, perceptive, and cognitive skills. In turn, this study will help further research on how peripheral vision training, faster reaction times, and improved conscious processing of visual information lead to better performance and higher levels of safety.

The limitations of the study were that given the heterogeneity of the participants, and the lack of an established visual training program for each sport it is necessary to further research that standardizes values and evaluations for each specific sport.

## 5. Conclusions

This study outlines the differences in visual skills between federated and non-federated athletes from several disciplines. The results have shown that federated athletes are more concerned about their ocular health. Nonetheless, no differences were found in the oculomotor skills of both groups. Therefore, further investigation is required to consider each sport discipline individually.

## Figures and Tables

**Table 1:** Demographic data of the study population.

	Total	Federated	Non-Federated	*p*-Value
No. of participants	52	18 (34.6%)	34 (65.4%)	
Age				0.116
Mean ± SD	22.7 ± 4.1	22.2 ± 4.2	23.0 ± 4.1	
Median [IQR]	21 [3]	21 [2]	22 [4]	
Gender				0.761
Male	34 (65.4%)	11 (32.4%)	23 (67.6%)	
Female	18 (34.6%)	7 (38.9%)	11 (61.1%)	

**Table 2:** Athletes regarding injuries or accidents give the answers.

	Total		Federated		Non-Federated		*p-*Value *	OR ** (95% CI)
	Yes	No	Yes	No	Yes	No		
Have you had a sports injury in the last year?	20 (38.5%)	32 (61.5%)	7 (38.9%)	11 (61.1%)	13 (38.2%)	21 (61.8%)	0.597	0.983 (0.479–2.019)
Have you ever suffered a concussion?	6 (11.5%)	46 (88.5%)	3 (16.7%)	15 (83.3%)	3 (8.8%)	31 (91.2%)	0.339	0.529 (0.119–2.360)
Have you ever had a severe fall or been injured in a traffic accident?	6 (11.5%)	46 (88.5%)	2 (11.1%)	16 (88.9%)	4 (11.8%)	30 (88.2%)	0.661	1.059 (0.214–5.236)
Do you have any chronic diseases? Or have you ever suffered from a severe disease?	6 (11.5%)	46 (88.5%)	2 (11.1%)	16 (88.9%)	4 (11.8%)	30 (88.2%)	0.661	1.059 (0.214–5.236)

* The Fisher exact test was used; ** The Odds ratio (and its 95% confidence interval) when answering “yes” to each of the questions for non-federated athletes, in comparison to federated athletes.

**Table 3:** Athletes, regarding their sports performance, give the answers.

	Total		Federated		Non-Federated		*p-*Value *	OR ** (95% CI)
	Yes	No	Yes	No	Yes	No		
Do you do any visual warm-up exercises?	2 (3.8%)	50 (96.2%)	1 (5.6%)	17 (94.4%)	1 (2.9%)	33 (97.1%)	0.577	0.529 (0.035–7.975)
Do you have any problems with balance?	1 (1.9%)	51 (98.1%)	0 (0.0%)	18 (100.0%)	1 (2.9%)	33 (97.1%)	0.654	-
Is your overall sports performance as consistent as you would like it to be?	26 (50.0%)	26 (50.0%)	14 (77.8%)	4 (22.2%)	12 (35.3%)	22 (64.7%)	**0.004**	**0.454** (**0.270**–**0.762**)
Is your level of sporting performance consistent throughout the game?	29 (55.8%)	23 (44.2%)	13 (72.2%)	5 (27.8%)	16 (47.1%)	18 (52.9%)	0.073	0.652 (0.412–1.029)
Does your performance decrease when you are under pressure?	18 (34.6%)	34 (65.4%)	6 (33.3%)	12 (66.7%)	12 (35.3%)	22 (64.7%)	0.569	1.059 (0.478–2.348)
Does your performance increase when you are under pressure?	33 (63.5%)	19 (36.5%)	11 (61.1%)	7 (38.9%)	22 (64.7%)	12 (35.3%)	0.515	1.059 (0.679–1.651)
Does your performance vary when you train indoors rather than outdoors?	17 (32.7%)	35 (67.3%)	4 (22.2%)	14 (77.8%)	13 (38.2%)	21 (61.8%)	0.196	1.721 (0.656–4.512)
Does your performance decrease when there are shadows on the playing field?	8 (15.4%)	44 (84.6%)	2 (11.1%)	16 (88.9%)	6 (17.6%)	28 (82.4%)	0.426	1.588 (0.356–7.081)

* The Fisher exact test was used; ** The Odds ratio (and its 95% confidence interval) when answering “yes” to each of the questions for non-federated athletes, in comparison to federated athletes. Significant *p*-values and OR (*p* < 0.05) are shown in bold.

**Table 4:** Athletes’ answers regarding vision.

	Total		Federated		Non-Federated		*p-*Value *	OR ** (95% CI)
	Yes	No	Yes	No	Yes	No		
Do you wear glasses to drive, play sports, watch TV, read, etc.?	18 (34.6%)	34 (65.4%)	4 (22.2%)	14 (77.8%)	14 (41.2%)	20 (58.8%)	0.144	1.853 (0.714–4.806)
Do you think that wearing glasses or contact lenses is a comfortable option when participating in your specific sport?	17 (60.7%)	11 (39.3%)	6 (66.7%)	3 (33.3%)	11 (57.9%)	8 (42.1%)	0.493	0.868 (0.476–1.583)
Do you suffer from intermittent blurred vision at far or near distances?	10 (19.2%)	42 (80.8%)	0 (0.0%)	18 (100.0%)	10 (29.4%)	24 (70.6%)	**0.008**	-
Do you suffer from red eyes?	5 (9.6%)	47 (90.4%)	1 (5.6%)	17 (94.4%)	4 (11.8%)	30 (88.2%)	0.428	2.118 (0.255–17.564)
Do you suffer from watery or itchy eyes?	8 (15.4%)	44 (84.6%)	1 (5.6%)	17 (94.4%)	7 (20.6%)	27 (79.4%)	0.153	3.706 (0.494–27.822)
Do you suffer from eye fatigue?	9 (17.3%)	43 (82.7%)	3 (16.7%)	15 (83.3%)	6 (17.6%)	28 (82.4%)	0.625	1.059 (0.300–3.743)
Do you suffer from headaches around the forehead, temple, or eyes?	16 (30.8%)	36 (69.2%)	6 (33.3%)	12 (66.7%)	10 (29.4%)	24 (70.6%)	0.504	0.882 (0.383–2.035)
Do you experience nausea associated with visual tasks?	2 (3.8%)	50 (96.2%)	1 (5.6%)	17 (94.4%)	1 (2.9%)	33 (97.1%)	0.577	0.529 (0.035–7.975)
Do you see flashes or halos around lights?	4 (7.7%)	48 (92.3%)	0 (0.0%)	18 (100.0%)	4 (11.8%)	30 (88.2%)	0.171	-
Do you experience double vision when looking at both near and far objects?	1 (1.9%)	51 (98.1%)	0 (0.0%)	18 (100.0%)	1 (2.9%)	33 (97.1%)	0.654	-
Do you need to squint or close one eye?	8 (15.4%)	44 (84.6%)	0 (0.0%)	18 (100.0%)	8 (23.5%)	26 (76.5%)	**0.024**	-
Are you sensitive to natural light, artificial light, and/or glare?	18 (34.6%)	34 (65.4%)	6 (33.3%)	12 (66.7%)	12 (35.3%)	22 (64.7%)	0.569	1.059 (0.478–2.348)
Do you use sunglasses? If you do, do these help you to increase your sports performance?	17 (32.7%) 5 (26.3%)	35 (67.3%) 14 (73.7%)	6 (33.3%) 2 (28.6%)	12 (66.7%) 5 (71.4%)	11 (32.4%) 3 (25.0%)	23 (67.6%) 9 (75.0%)	0.590 0.634	0.971 (0.430–2.191) 0.875 (0.190–4.030)

* The Fisher exact test was used; ** The Odds ratio (and its 95% confidence interval) when answering “yes” to each of the questions for non-federated athletes, in comparison to federated athletes. Significant *p*-values and OR (*p* < 0.05) are shown in bold.

**Table 5:** Visual assessment results.

	Total			Federated			Non-Federated			*p-*Value	Difference ± SE (95% CI) *
	n	Mean ± SD	Median [IQR]	n	Mean ± SD	Median [IQR]	n	Mean ± SD	Median [IQR]		
Static visual acuity Monocular VA (RE) Monocular VA (LE) Binocular VA	52	1.10 ± 0.22 1.10 ± 0.21 1.18 ± 0.16	1.20 [0.29] 1.20 [0.25] 1.26 [0.06]	18	1.16 ± 0.20 1.20 ± 0.09 1.22 ± 0.12	1.26 [0.11] 1.26 [0.11] 1.26 [0.01]	34	1.07 ± 0.23 1.05 ± 0.24 1.16 ± 0.17	1.20 [0.37] 1.15 [0.36] 1.26 [0.14]	0.079 **0.010** 0.310	0.09 ± 0.08 (−0.11–0.21) **0.15 ± 0.07 (0.02–0.31**) 0.06 ± 0.04 (−0.05–0.12)
Dynamic visual acuity Monocular VA (RE) Monocular VA (LE) Binocular VA % correct answers (RE) % correct answers (LE) % correct answers binocular	51	1.10 ± 0.59 1.07 ± 0.83 0.87 ± 0.19 76.27 ± 21.91 68.82 ± 32.90 90.39 ± 13.71	0.89 [0.35] 0.86 [0.47] 0.80 [0.10] 80.00 [20] 80.00 [50] 100.00 [20]	18	0.99 ± 0.46 0.88 ± 0.48 0.86 ± 0.13 83.33 ± 20.58 73.89 ± 32.92 92.78 ± 12.27	0.85 [0.28] 0.83 [0.30] 0.80 [0.12] 90.00 [30] 90.00 [50] 100.00 [13]	33	1.15 ± 0.66 1.17 ± 0.96 0.88 ± 0.22 72.42 ± 21.94 66.06 ± 33.07 89.09 ± 14.44	0.96 [0.42] 0.87 [0.77] 0.80 [0.10] 80.00 [30] 70.00 [55] 90.00 [20]	0.190 0.385 0.992 **0.040** 0.383 0.278	−0.16 ± 0.22 (−0.62–0.32) −0.29 ± 0.21 (0.64–0.24) −0.02 ± 0.06 (−0.16- 0.09) **10.91 ± 8.33 (−9.24–25.91)** 7.83 ± 12.72 (−16.28–37.39) 3.69 ± 4.93 (−4.85–15.96)
Contrast sensitivity 1.5 3 6 12 18	50	133.56 ± 32.41 196.84 ± 44.59 230.06 ± 54.95 115.88 ± 52.83 49.02 ± 39.11	150.00 [47] 210.00 [0] 250.00 [0] 150.00 [89] 50 [93]	17	141.71 ± 18.47 210.00 ± 0.00 250.00 ± 0.00 141.82 ± 33.71 67.00 ± 39.08	150.00 [0] 210.00 [0] 250.00 [0] 150.00 [0] 100 [62]	33	129.36 ± 37.21 190.06 ± 53.89 219.79 ± 65.60 102.52 ± 56.24 39.76 ± 36.32	150.00 [47] 210.00 [0] 250.00 [0] 150.00 [100] 26.00 [43]	0.285 0.095 **0.043** **0.008** **0.028**	12.35 ± 9.70 (−11,74–29.39) 19.94 ± 7.65 (−8.56–23.86) **30.21 ± 9.03 (−6.97–31.32)** **39.30 ± 17.90 (0.64–76.54)** **27.24 ± 16.15 (−5.66–62.83)**
Fixation disparity (prismatic diopters)	52	−0.00 ± 0.09	−0.13 [0.12]	18	−0.01 ± 0.07	−0.02 [0.09]	34	0.00 ± 0.10	−0.01 [0.43]	0.939	−0.01 ± 0.02 (−0.06–0.05)
Stereopsis	39	67.18 ± 43.65	40.00 [40]	13	58.46 ± 32.11	40.00 [30]	26	71.54 ± 48.39	40.00 [70]	0.691	−13.08 ± 4.44 (−14.69–5.80)
Visual memory Correct answers (%) Average time (s)	52	97.18 ± 5.17 1.51 ± 0.37	100.00 [6.67] 1.48 [0.40]	18	97.41 ± 5.18 1.46 ± 0.24	100 [1.67] 1.48 [0.37]	34	97.06 ± 5.24 1.53 ± 0.43	100.00 [6.67] 1.46 [0.40]	0.684 0.969	0.35 ± 1.69 (−2.83–4.32) −0.07 ± 0.10 (−0.28–0.14)
Identification Correct answers (%) Average time (s)	51	91.72 ± 5.45 545.65 ± 123.52	93.59 [5.00] 557.76 [86.96]	18	92.32 ± 6.50 570.00 ± 56.93	95.00 [6.25] 577.27 [101.95]	33	91.38 ± 4.87 532.36 ± 146.97	92.50 [4.90] 557.22 [80.84]	0.123 0.608	0.94 ± 2.33 (−3.28–6.60) 37.64 ± 11.99 (−36.41–14.44)
Anticipation time Correct answers (%) Average time (s)	52	20.58 ± 17.31 0.14 ± 0.20	20.00 [20] 0.07 [0.08]	18	24.44 ± 17.56 0.10 ± 0.09	20.00 [13] 0.08 [0.09]	34	18.53 ± 17.08 0.16 ± 0.24	20.00 [33] 0.07 [0.08]	0.207 0.483	5.91 ± 6.63 (−5.65–22.32) −0.06 ± 0.05 (−0.19–0.05)
Peripheral awareness Correct answers (%) Average time (s) Correct answers right side (%) Correct answers left side (%)	48	52.73 ± 14.06 1.40 ± 0.31 55.21 ± 25.76 48.61 ± 20.29	50.00 [18.75] 1.32 [0.36] 50.00 [33.33] 50.00 [33.33]	17	51.84 ± 11.00 1.41 ± 0.33 54.90 ± 26.20 45.10 ± 19.33	50.00 [15.63] 1.40 [0.42] 50.00 [41.67] 50.00 [33.33]	31	53.23 ± 15.64 1.39 ± 0.31 55.38 ± 25.96 50.54 ± 20.85	50.00 [25] 1.29 [0.36] 50.00 [33.33] 50.00 [33.33]	0.907 0.880 0.974 0.427	−1.39 ± 5.96 (−14.65–10.48) 0.02 ± 0.11 (−0.24–0.24) −0.48 ± 8.12 (−16.26–18.34) −5.44 ± 7.75 (−23.82–9.23)
Hand-eye coordination Correct answers (%) Average time (s)	52	81.81 ± 20.79 0.59 ± 0.13	88.00 [27] 0.61 [0.10]	18	82.67 ± 17.84 0.58 ± 0.10	90.00 [33] 0.59 [0.12]	34	81.35 ± 22.44 0.59 ± 0.15	88.00 [21] 0.61 [0.10]	0.884 0.863	1.32 ± 7.33 (−12.14–18.80) −0.01 ± 0.04 (−0.07–0.12)

* The difference ± the standard error and the 95% confidence interval are calculated as the mean of the federated participants minus the mean of the non-federated participants. Significant differences (*p* < 0.05) are marked in bold. RE: Right eye; LE: Left eye; s: second; SD: Standard deviation; IQR: Interquartile range; CI: Confidence Interval.

## Data Availability

Not applicable.
